# Exercise, character strengths, well-being, and learning climate in the prediction of performance over a 6-month period at a call center

**DOI:** 10.3389/fpsyg.2014.00497

**Published:** 2014-06-23

**Authors:** Saleh Moradi, Ali A. Nima, Max Rapp Ricciardi, Trevor Archer, Danilo Garcia

**Affiliations:** ^1^Department of Management, University of OtagoDunedin, New Zealand; ^2^Network for Empowerment and Well-BeingGothenburg, Sweden; ^3^Department of Psychology, University of GothenburgGothenburg, Sweden; ^4^Institute of Neuroscience and Physiology, Centre of Ethics, Law and Mental Health, University of GothenburgGothenburg, Sweden

**Keywords:** call center, character strengths, learning climate, performance, psychological well-being, subjective well-being, virtues

## Abstract

**Background:** Performance monitoring might have an adverse influence on call center agents' well-being. We investigate how performance, over a 6-month period, is related to agents' perceptions of their learning climate, character strengths, well-being (subjective and psychological), and physical activity.

**Method:** Agents (*N* = 135) self-reported perception of the learning climate (Learning Climate Questionnaire), character strengths (Values In Action Inventory Short Version), well-being (Positive Affect, Negative Affect Schedule, Satisfaction With Life Scale, Psychological Well-Being Scales Short Version), and how often/intensively they engaged in physical activity. Performance, “time on the phone,” was monitored for 6 consecutive months by the same system handling the calls.

**Results:** Performance was positively related to having opportunities to develop, the character strengths clusters of Wisdom and Knowledge (e.g., curiosity for learning, perspective) and Temperance (e.g., having self-control, being prudent, humble, and modest), and exercise frequency. Performance was negatively related to the sense of autonomy and responsibility, contentedness, the character strengths clusters of Humanity and Love (e.g., helping others, cooperation) and Justice (e.g., affiliation, fairness, leadership), positive affect, life satisfaction and exercise Intensity.

**Conclusion:** Call centers may need to create opportunities to develop to increase agents' performance and focus on individual differences in the recruitment and selection of agents to prevent future shortcomings or worker dissatisfaction. Nevertheless, performance measurement in call centers may need to include other aspects that are more attuned with different character strengths. After all, allowing individuals to put their strengths at work should empower the individual and at the end the organization itself. Finally, physical activity enhancement programs might offer considerable positive work outcomes.

## Introduction

Amongst various organizational factors contributing to workers' well-being, performance monitoring has received less attention in prior studies (Holman et al., 2002). Stanton (2000) defines performance monitoring as those practices that involve “the observation, examination, and/or recording of employee work-related behaviors, with and without technological assistance” (p. 87). Utilizing performance monitoring, the first benefit that comes to mind is being able to monitor and improve employee performance, which ensure cost efficiency and customer satisfaction (Alder, 1998). Yet, employees are believed to profit from performance monitoring by means of the feedback they can obtain from their own performance; the feedback brings about an opportunity for employees to recognize their development potentials, improve their performance, and even feel more satisfied from the knowledge of their improved performance and abilities to cope better with work demands (Hackman and Oldham, 1976; Grant and Higgins, 1989; Aiello and Shao, 1993). Performance monitoring has been suggested even as a way to engender intrinsic motivation in employees and improve their well-being (Stanton, 2000). Nonetheless, performance monitoring has its own critics as it may adversely influence employees' remuneration and/or their relationship with coworkers (Alder, 1998). It has been known for a long time that performance monitoring can be used as an intermediary to intensify employees' workload and increase the level of work demand (Smith et al., 1992). Critics likewise distinguish performance monitoring as an influential factor on well-being, but mostly as a detrimental factor which impacts employees' well-being negatively (Stanton, 2000).

Call centers make perfect workplaces to study performance monitoring as most of them have electronic performance monitoring systems implemented to supervise their agents by means of several quantitative indicators such as length of call, number of calls, and amount of time on the phone. The quality of calls is, sometimes, assessed by listening or recording overtly or without the agent's knowledge (Taylor and Bain, 1999)—although the agents always know of the possibility of being recorded. Most often, the quantitative data generated from measuring seconds of employees' work/rest moments is the only way used to assess their performance, and even to determine their incentives and remuneration (Taylor and Bain, 1999; Holman et al., 2002; Garcia and Archer, 2012). This may explain why call centers are sometimes called “electronic panopticons” (Fernie and Metcalf, 1998, p. 9), “electronic sweatshops” or “the dark satanic mills of the twenty-first century” (Holman, 2003a, p. 123).

From an organizational point of view call centers are the frontline actors of the organization to deal with customer inquiries, hear their voices, and are representing the way an organization values its customers. Every second that an agent is not on the phone amounts to the precious queue time for customers (Garcia et al., 2012b). An appropriate workplace should be able to promote employees' satisfaction and well-being, and call centers are not an exception of this axiom, (Wegge et al., 2006). Prior studies, however, show that work is a demanding and stressful experience for many call center agents (Holman, 2005). Call centers' monitoring systems have shown to consign work-related stress, which in turn can possibly decreases employees' well-being and lessen their job satisfaction and organizational commitment (Taylor and Bain, 1999; De Ruyter et al., 2001). The working conditions in call centers might also affect employees' opportunities to organize their own work, and diminish their sense of freedom for decision-making (Garcia and Archer, 2012). Finally, about 50% labor turnover in U.S. call centers in 2000 (Bordoloi, 2004), a 21% turnover rate in a study of 14 call centers in Switzerland (Baumgartner et al., 2002), and overall 30–50% estimated average turnover rate per year (IBISWorld, 2008) utter clearly about the situation this specific type of work design leads to.

Understandably, call center management methods is growing as the popular subject of many studies (Deery and Kinnie, 2004); and as Taylor and Bain (1999, p. 102) suggest: “call center managements face a plethora of problems concerning motivation and commitment, labor turnover, the effectiveness of supervision and the delivery of quality and quantity performance.” This study, accordingly, is devoted to take a comprehensive look at call center agents' performance. We want to investigate how performance over a 6-month period, monitored as “time on the phone,” is related to agents' perceptions of their learning climate, positive personal characteristics (i.e., character strengths), well-being (subjective and psychological well-being), and physical activity.

### Learning climate

The work climate denotes employees' perception of how they are treated and managed in their organization. Organizational climate can be defined as a set of attributes perceivable about an organization, which “may be deduced from the way that the organization and/or subsystems deal with their members and environment” (Hellriegel and Slocum, 1974, p. 256); or, as employees' shared perceptions of organization's policies, practices, and procedures and the behaviors supported, expected, and rewarded in the organization (Schneider et al., 2011). Moreover, organizational climate can be considered as the indicator of organizational culture as deeper and less consciously held perceptions and affections by members (Schein, 1985). Organizational climate comprises different aspects ranging from leadership style, work conditions, work force responsibilities and development opportunities, job requirements, and general satisfaction.

Moreover, individuals tend to cluster related facets of work settings and perceive it as a specific climate of their organization, or their organizations focused climate such as climate for safety or climate for service (Schneider and Reicher, 1983; Schneider et al., 2011). Similarly, organizations may develop a climate focused on learning. Learning climate has been described as a climate that actively encourages behaviors and practices pertained to continuous development (Honey and Mumford, 1996). Learning climate may include mission, vision, corporate goals and strategies, structures and practices supporting learning, shared vision and goals, cooperative learning, challenging attitudes, continuous improvement, management support, learning-to-learn skills and lifelong learning commitment (Malone, 2003). Bartram et al. (1993a,b) have developed a measurement instrument for learning climate, which denotes seven facets of climate as: (1) *Management Relations and Style*, reflecting leadership style; (2) *Time*, reflecting the amount of time available for members to perform their tasks and learn; (3) *Autonomy and Responsibility*, denoting the control level and possibilities for decision-making and initiating actions; (4) *Team Style*, which is reflecting the possibilities for learning from senior and proficient colleagues; (5) *Opportunity to Develop*, comprising opportunities to learn new skills within the same job and probable job rotation strategies; (6) *Guidelines on How to Do the Job*, reflecting availability of task instructions and guidelines; (7) *Contentedness*, reflecting general satisfaction with regard to the workplace (Bartram et al., 1993a,b, 1996).

Organizations quality of their learning climate is usually assumed to be influential in the rate of organizational learning and organizational performance (Moss-Kanter, 1983). Organizational learning is an outcome of employees' attempts to deal with issues and problems they are experiencing at the workplace (Argyns and Schon, 1996). Accordingly, learning climate is believed to be important in organizations endeavor to motivate employees in order to enhance their efforts into their work (Neal et al., 2005; Boudrias et al., 2010). Creating a learning climate in which employees are able to learn from each other and new experiences is essential for the development of an organization and augmentation of well-being among its employees (Mikkelsen and Gronhaug, 1999; Sprigg and Jackson, 2006), that is, the empowerment of workers (Garcia and Archer, 2012).

Call-centers as specific workplaces where agents are spending most of their time on phone by themselves, responding to inquiries from customers may also hold a particular climate. Work at call center usually requires single and sometime monotonous work, thus, the influence of social aspects of the work climate (e.g., acting as a team member, helpful, and cooperative behavior) might not be applicable in such a context. Nonetheless, other learning climate facets (i.e., Management Relations and Style, Time, Autonomy and Responsibility, Guidelines on How to Do the Job, and Contentedness) in call centers may be more relevant. Hence, this study tries to understand the learning climate facets contributing in enhancement of call center agents' performance. This leads to our first research question (RQ):

*RQ1:* Which learning climate facets significantly predict higher performance over a 6-month period?

### Character strengths

Peterson and Seligman (2004) postulate that an important part of human functioning relates to strengths of character. Character components can be represented by values in action classification of strengths (Peterson and Seligman, 2004) as measurable continua of positive individual differences (McGrath et al., 2010). The values in action instrument classifies 24 character strengths into six main clusters called virtues: (1) *Wisdom and Knowledge*, cognitive strengths which require acquisition and use of knowledge such as creativity, curiosity, judgment, love of learning, and perspective; (2) *Courage*, strengths which involve willingness to achieve goals despite internal or external confrontation such as bravery, perseverance, honesty, and zest; (3) *Humanity and Love*, interpersonal strengths involving learning and supporting others such as capacity to love and be loved, kindness, and social intelligence; (4) *Justice*, strengths underling “healthy community life” such as teamwork, fairness, and leadership; (5) *Temperance*, as strengths which are preventing from actions beyond what is usual or proper such as forgiveness, modesty, prudence, and self-regulation; (6) *Transcendence*, strengths such as appreciation of beauty, gratitude, hope, humor, and religiousness (Seligman, 2002; Wright and Goodstein, 2007).

Peterson and Seligman (2004) consider an individual to possess, celebrate, and frequently exercise three to seven core strengths, so called “signature” strengths. The application of signature strengths in daily life has been suggested to contribute to an individual's life satisfaction, well-being, sense of flow, meaning in life, physical health and recovery from illness, and quality of life in general (Csikszentmihalyi, 1990; Seligman, 2002; Park et al., 2004; Seligman et al., 2005; Peterson, 2006; Littman-Ovadia and Steger, 2010; Proctor et al., 2011). In other words, frequently using one's signature strengths of character leads to the empowerment of the individual. Organizational studies, for instance, propose that endorsement and deployment of signature strengths in workplace enhances overall positive experiences about the working environment (Littman-Ovadia and Steger, 2010; Harzer and Ruch, 2012). Organizations can gain from the application of strengths under these two conditions; firstly, aptitude of employees to show behaviors related to a specific strength relies on a certain level that individuals need to possess that strength; secondly, organizational circumstances (e.g., work climate, work design) have to let or demand the expression of the strength (Harzer and Ruch, 2012). Moreover, different working positions tend to endorse some strengths more than others (e.g., managing positions endorse leadership and courage). Previous studies emphasize that benefits of applying character strengths can only be flourished when being able to use one's signature strengths in significant life domains (Duckworth et al., 2005); accordingly, providing opportunities for employees to deploy their signature strengths in their work life is a key factor in workplace engagement, which in turn results in variety of work-related outcomes (e.g., enhanced performance; Harter et al., 2002).

Call-centers, due to their specific work design, may call for particular character strengths to boost their outcomes. Interpersonal strengths (i.e., love, kindness, and social intelligence) are probably not helpful when an agent is giving financial advice to a customer with other customers on hold to be answered in the shortest time, this might also attenuate teamwork as well. All said, what happens when an agent denote social intelligence, humor, and creativity as her core character strengths, considering that call centers work design more often demands fast, short, impersonal, and standardized, and even pre-determined in some cases, responses to customers? Which character strengths are more prone to foster enhanced performance for a call center agent? These are some of the uncertainties that lead to our second research question:

*RQ2:* Which main types of character strengths clusters (i.e., virtues) significantly predict performance over a 6-month period?

### Well-being and exercise

Well-being denotes “the state of being happy, healthy, or prosperous” (Cloninger, 2004; Well-being, 2013), hence comprising both physical and psychological state of individuals. According to Seaward (1994), well-being significantly relies on the balance between individual's physical, emotional, intellectual and spiritual aspects (see also Cloninger, 2004). Well-being has been studied from two distinctive viewpoints (Ryan and Deci, 2001; Kjell et al., 2013; Garcia et al., 2014a). First, studies of so called “subjective well-being” (Diener, 1984) focus on assessment of individual's judgments of life satisfaction, the frequency of positive affect, and the infrequency of negative affect (hedonic point of view); second, “psychological well-being” (Ryff, 1989) studies (eudemonic point of view) which focus on both theoretical and operational aspects of well-being by including six distinct constructs of well-being in their studies (i.e., autonomy, personal growth, self-acceptance, life purpose, environmental mastery, and positive relations with others). Psychological well-being constructs identify what promotes effective adoption to life events and emotional and physical health (Ryff, 1989; for a review see Garcia et al., 2012a).

Work environment has been clearly related to individuals' perception of both physical and psychological health (Sutherland and Cooper, 2000), and working in a comfortable and supportive environment enhances well-being among individuals (McGuire and McLaren, 2009). Previous studies have suggested that while physical enhancement of work environments will increase productivity of work forces (Brill, 1992), stressful work environments, in turn, result in physical and mental ill-health symptoms and low job satisfaction (Cunha and Cooper, 2002). Call-centers have been considered as one of the workplaces where agents experiencing both unpleasant physical and mental working conditions, considering the fact that a call center work tasks are often “sedentary and one-sided in front of the computer most of the day” (Norman et al., 2004, p. 55). In many call centers the operators do not possess their own work unit, and unavailability of ergonomic and optimal workstations (e.g., adjustable chairs, tables, keyboard placements, input device placement, etc.) cause reports of pain from operators at the end of working days (Norman et al., 2004). Moreover, musculoskeletal disorders seem to be relatively common in call centers (Hales et al., 1994; Halford and Cohen, 2003; Norman et al., 2004). Halford and Cohen (2003) argued that performance-monitoring, workload, particular management-worker relations (e.g., lack of support), work-related stress, job characteristics (e.g., call-handling, repetition, monotony and noise-levels), lack of job control and frequency of computer usage are significantly associated with musculoskeletal disorder symptoms. Health issues have also been reported among call center agents due to time pressure, duration of the shifts (Ferreira et al., 1997), and work-related stress caused by shift work, lack of control and support at workplaces (Fenety et al., 1999). Furthermore, excessive use of scripts has been criticized because of reducing the skills of agents and their need to think (Wilson, 2006), and its positive relation to emotional exhaustion (Holman, 2003a,b). In a recent study, Krause et al. (2010) found a significant relation between effort-reward imbalance in call centers with musculoskeletal disorders among employees controlling for duration of computer use, ergonomic workstation design, physical activities during leisure time and other individual worker characteristics.

In recent years, however, studies have suggested physical activities (e.g., training programs) to be an efficient treatment for work-related health issues. Regular physical exercise involves planned, structured physical activity in order to improve aspects of physical fitness and functional capacity (Morris and Schoo, 2004). Regular physical activities have been positively associated with an individual's higher levels of subjective well-being and psychological well-being, improved coping, less depression, anger, and stress, better fitness, higher levels of sense of coherence, stronger feeling of social integration, improved physical self-concept, less psychosomatic complaints and musculoskeletal disorder discomfort, and reduced levels of mental fatigue (Norris et al., 1992; Alfermann and Stoll, 2000; Hassmén et al., 2000; Norlander et al., 2002; Lacaze et al., 2010; Garcia et al., 2012a).

Some detriments of working in call centers seem to ameliorate by employees regular exercising. Yet, Renton et al. (2011) noticed that call center employers, despite their motivation to promote physical activity among employees, have concerns regarding participation, fairness and cost and special limitations of workplaces. Interestingly, employers put forward the nature of call center work as one of the barrier for promoting physical activity among their employees (Renton et al., 2011). Considering the significance of well-being notions among call center agents and its above mentioned positive effects both on individual and organizational level, this study tries to investigate possible relations between well-being aspects and exercise with agents' performance. This leads to our final research questions:

*RQ3:* Does well-being (i.e., positive affect, negative affect, life satisfaction, and psychological well-being) significantly predict higher performance level over a 6-month period?

*RQ4:* Does exercise frequency and/or intensity significantly predict higher performance level over a 6-month period?

## Methods

### Participants and procedure

At Time 1 (T1) agents from a call center (135) in Sweden were invited to self-report their perception of the learning climate, virtues and character strengths, well-being, and how often and how intensively they engaged in physical activity. All agents were informed that their participation was voluntary and confidential and no supervisors were invited to participate. The job of the agents at this specific call center was to answer questions regarding financial advice. Agents were instructed to provide their “worker number” in order to trace responses from the T1 and T2. All agents participated in the first part of the Study and received cinema tickets for their collaboration. Participants' performance was then assessed for the next 6 consecutive months by the same system handling the calls. At the end of the 6 months, participants were asked to retrieve their performance and to report it directly to one of the researchers along their “worker number.” Agents who provided their performance at the second part of the study received a cinema ticket for their collaboration. Although all agents participated in T1, a total of 110 agents (mean age = 42.77 *SD* = 13.35, 84 females and 26 males) chose to participate in T2.

### Measures

#### Learning climate

The Learning Climate Questionnaire (Bartram et al., 1993a,b) comprises 70 items (1 = *extremely disagree*, 5 = *extremely agree*), organized in seven subscales that provide means for looking at the working climate in more detail: Management Relations and Style (e.g., “My immediate manager makes me feel like a valuable member of the team”), Time (e.g., “I have time to do my job properly”), Autonomy and Responsibility (e.g., “I feel free to organize my work the way I want to”), Team Style (e.g., “If we ask each other for help it is given”), Opportunities to Develop (e.g., “There are lots of different ways to learn new jobs here”), Guidelines on How to Do the Job (e.g., “Information relevant to my job is kept up-to-date”), and Contentedness (e.g., “People tend to put each other down,” reversed item).

#### Character strengths

The short version of the Values In Action Inventory (Seligman, 2002) measures strengths of character that are organized in 6 character strengths clusters or virtues: Wisdom and Knowledge (e.g., “I am always curious about the world”), Courage (e.g., “I have taken frequent stands in the face of strong opposition”), Humanity and Love (e.g., “I have voluntary helped a neighbor/colleague in the last month”), Justice (e.g., “I work best when I am in a group”), Temperance (“I control my emotions”), Transcendence (e.g., “In the last month, I have been thrilled by excellence in music, art, drama, film, sport, science, or mathematics”). The participants are instructed to address grade of agreement in a 5-point Likert scale (1 = *very much unlike me*, 5 = *very much like me*).

#### Subjective well-being

For the measuring affective component of subjective well-being we used the Positive Affect and Negative Affect Schedule (Watson et al., 1988), which requires participants to indicate on 5-point Likert scale to what extent (1 = *very slightly*, 5 = *extremely*) they generally experienced 20 different adjectives within the last few weeks. The positive affect scale includes 10 adjectives such as strong, proud, and interested; and the negative affect scale includes 10 adjectives such as afraid, ashamed, and nervous. The cognitive component of subjective well-being was measured using the Satisfaction With Life Scale (Diener et al., 1985), which consists of 5 items (e.g., “In most of my ways my life is close to my ideal”) that require a response on a 7-point Likert scale (1 = *strongly disagree*, 7 = *strongly agree*). The Swedish versions of these instruments have been used in published studies (e.g., Garcia et al., 2012a).

#### Psychological well-being

We used the short version of the Scales of Psychological Well-Being (the short version; Clarke et al., 2001), which comprises 18 items; 3 items for each of the 6 psychological well-being dimensions. These dimensions are: (1) positive relations with others (e.g., “People would describe me as a giving person, willing to share my time with others”), (2) environmental mastery (e.g., “ I am quite good at managing the responsibilities of my daily life”), (3) self-acceptance (e.g., “I like most aspects of my personality”), (4) autonomy (e.g., “I have confidence in my own opinions, even if they are contrary to the general consensus”), (5) personal growth (e.g., “For me, life has been a continuous process of learning, changing, and growth”), and (6) purpose in life (e.g., “Some people wander aimlessly through life, but I am not one of them”). The Swedish version has been used in previous studies (e.g., Nima et al., 2013) and in the current study the total psychological well-being score (i.e., the sum of the 18 items) was used.

#### Exercise frequency and intensity

Participants were asked to report how frequent (1 = *seldom or never*, 5 = *Very often*) and how intensive (1 = *low intensity*, 10 = *very intensive*) they engaged in physical activity. These two questions were imbedded among the age and gender questions. These questions have been validated to produce reliable answers regarding individuals' propensity to exercise (Karlsson and Archer, 2007).

#### Performance

Each worker's performance was assessed by the same system handling the calls each day over a 6-month period. Basically each worker has a minimum of 5 h schedule each day for being logged in the system waiting and handling inbound- and outbound phone calls (i.e., “time on the phone”). The system monitors these actions and divides the accumulated “time on the phone” by the time the agent was originally schedule to be on the phone. In other words, the performance measure is a percentage of the time the organization expects the agents to be working on calls or being ready to receive calls and the actual time agents deliver. The system handles absenteeism, caused by sickness or other type of absenteeism accepted by the organization, by simply not taking those days or hours into account when the performance measure is computed. This measure of performance is widely used in call centers (e.g., Garcia and Archer, 2012; Garcia et al., 2012b).

### Statistical treatment

Expectation-Maximization Algorithm was used for handling and imputing missing data. *Little's Chi-Square* test for Missing Completely at Random was, χ2_(306, *n* = 110)_ = 334.07, *p* = 0.13. To reduce the impact of variables with outliers we first standardized the scores of each variable and tested if any cases had larger standardized scores than ±3.29, as recommended by Tabachnick and Fidell (2007). The analysis detected seven cases as outliers in the performance variable (i.e., standardized scores in excess of ±3.29). These scores were changed to the next highest/lowest (non-outlier) number +1/−1 (see Tabachnick and Fidell, 2007, p. 77). This reduced skewness for performance at work from −1.5 to −0.59 and kurtosis from 5.08 to.18.

## Results

The correlations, means, standard deviations, and reliability coefficients are reported in Table 1. Correlation analysis demonstrates that Time, Autonomy and Responsibility, and Contentedness together with the character strength cluster of Temperance are the only variables that correlate with Performance.

**Table 1:** **Correlations among variables in the study (*N* = 110)**.

	**1**	**2**	**3**	**4**	**5**	**6**	**7**	**8**	**9**	**10**	**11**	**12**	**13**	**14**	**15**	**16**	**17**	**18**	**19**	**20**
Performance (1)																				
Management relations and style (2)	0.00																			
Time (3)	−0.22^*^	0.24^*^																		
Autonomy and responsibility (4)	−0.29^**^	0.44^**^	0.65^**^																	
Team style (5)	−0.09	0.38^**^	0.43^**^	0.43^**^																
Opportunities to develop (6)	−0.07	0.55^**^	0.48^**^	0.68^**^	0.41^**^															
Guidelines on how to do the job (7)	−0.11	0.57^**^	0.61^**^	0.67^**^	0.63^**^	0.69^**^														
Contentedness (8)	−0.29^**^	0.22^*^	0.45^**^	0.41^**^	0.38^**^	0.45^**^	0.45^**^													
Wisdom and knowledge (9)	0.11	0.15	0.26^**^	0.30^**^	0.26^**^	0.30^**^	0.35^**^	0.11												
Courage (10)	0.17	0.33^**^	0.32^**^	0.23^*^	0.23^*^	0.19^*^	0.34^**^	−0.04	0.48^**^											
Humanity and love (11)	0.05	0.21^*^	−0.01	0.04	0.24^*^	0.24^*^	0.19	−0.01	0.38^**^	0.33^**^										
Justice (12)	−0.14	0.17	0.19^*^	0.24^*^	0.31^**^	0.33^**^	0.27^**^	0.14	0.57^**^	0.23^*^	0.52^**^									
Temperance (13)	0.34^**^	0.15	0.00	−0.08	0.12	0.01	0.04	0.05	0.10	0.23^*^	0.29^**^	0.13								
Transcendence (14)	0.02	0.41^**^	0.25^**^	0.39^**^	0.38^**^	0.49^**^	0.45^**^	0.15	0.55^**^	0.40^**^	0.57^**^	0.64^**^	0.15							
Positive affect (15)	−0.14	0.40^**^	0.42^**^	0.54^**^	0.44^**^	0.56^**^	0.58^**^	0.26^**^	0.48^**^	0.36^**^	0.28^**^	0.40^**^	0.09	0.54^**^						
Negative affect (16)	0.02	−0.30^**^	−0.22^*^	−0.30^**^	−0.23^*^	−0.25^**^	−0.31^**^	−0.30^**^	−0.07	−0.20^*^	−0.17	−0.12	−0.08	−0.18	−0.23^*^					
Life satisfaction (17)	−0.16	0.35^**^	0.25^**^	0.36^**^	0.37^**^	0.36^**^	0.32^**^	0.26^**^	0.27^**^	0.15	0.31^**^	0.38^**^	0.22^*^	0.55^**^	0.43^**^	−0.23^*^				
Psychological well-being (18)	0.08	0.31^**^	0.37^**^	0.34^**^	0.30^**^	0.32^**^	0.36^**^	0.19	0.61^**^	0.52^**^	0.42^**^	0.44^**^	0.15	0.63^**^	0.51^**^	−0.30^**^	0.49^**^			
Exercise frequency (19)	0.14	−0.15	0.01	0.04	−0.06	−0.07	0.00	0.09	0.29^**^	0.07	0.17	0.21^*^	−0.03	0.18	0.08	0.01	0.15	0.19^*^		
Exercise intensity (20)	−0.11	−0.05	0.17	0.01	−0.12	−0.09	−0.08	0.05	0.24^*^	0.10	−0.11	0.10	−0.09	0.05	−0.03	−0.01	0.06	0.18	0.29^**^	
Mean	83.31	4.12	3.22	3.27	4.18	3.12	3.78	2.87	7.21	7.34	7.49	6.68	6.70	6.91	3.66	1.53	4.99	4.59	4.48	6.17
Sd.	16.22	0.62	0.80	0.64	0.44	0.61	0.50	0.64	0.83	0.98	1.40	1.16	0.87	0.94	0.64	0.47	1.14	0.52	1.06	1.72
Cronbach' α	–	0.87	0.90	0.81	0.77	0.80	0.73	0.81	0.65	0.47	0.55	0.67	0.94	0.73	0.89	0.80	0.88	0.75	–	–

* p < 0.05;

** p < 0.01;

^***^p < 0.001.

### Regression analysis

A Multiple Regression Analysis was conducted to assess whether learning climate, character strengths, subjective well-being, psychological well-being, Exercise Frequency and Exercise Intensity uniquely predicted performance at work among agents. The model explained 55.4% of the variance in performance at work [*F*_(19, 109)_ = 5.89, *p* < 0.001]. There were positive associations between performance at work and Opportunities to Develop (*B* = 13.23, β = 0.50, *t* = 3.97, *p* < 0.001), Wisdom and Knowledge (*B* = 5.15, β = 0.26, *t* = 2.30, *p* = 02), Temperance (*B* = 7.17, β = 0.39, *t* = 4.91, *p* < 0.001), and Exercise Frequency (*B* = 5.29, β = 0.35, *t* = 4.21, *p* < 0.001). There were negative associations between performance at work and Autonomy and Responsibility (*B* = −11.49, β = −0.45, *t* = −3.67, *p* < 0.001), Contentedness (*B* = −9.66, β = −0.38, *t* = −4.12, *p* < 0.001), Humanity and Love (*B* = −3.33, β = −0.28, *t* = −2.86, *p* = 0.005), Justice (*B* = −4.56, β = −0.32, *t* = −3.12, *p* = 0.002), Positive Affect (*B* = −5.86, β = −0.23, *t* = −2.27, *p* = 0.03), Life Satisfaction (*B* = −3.54, β = −0.24, *t* = −2.57, *p* = 0.01) and Exercise Intensity (*B* = −1.88, β = −0.20, *t* = −2.38, *p* = 0.02). See Table 2 for the details.

**Table 2:**
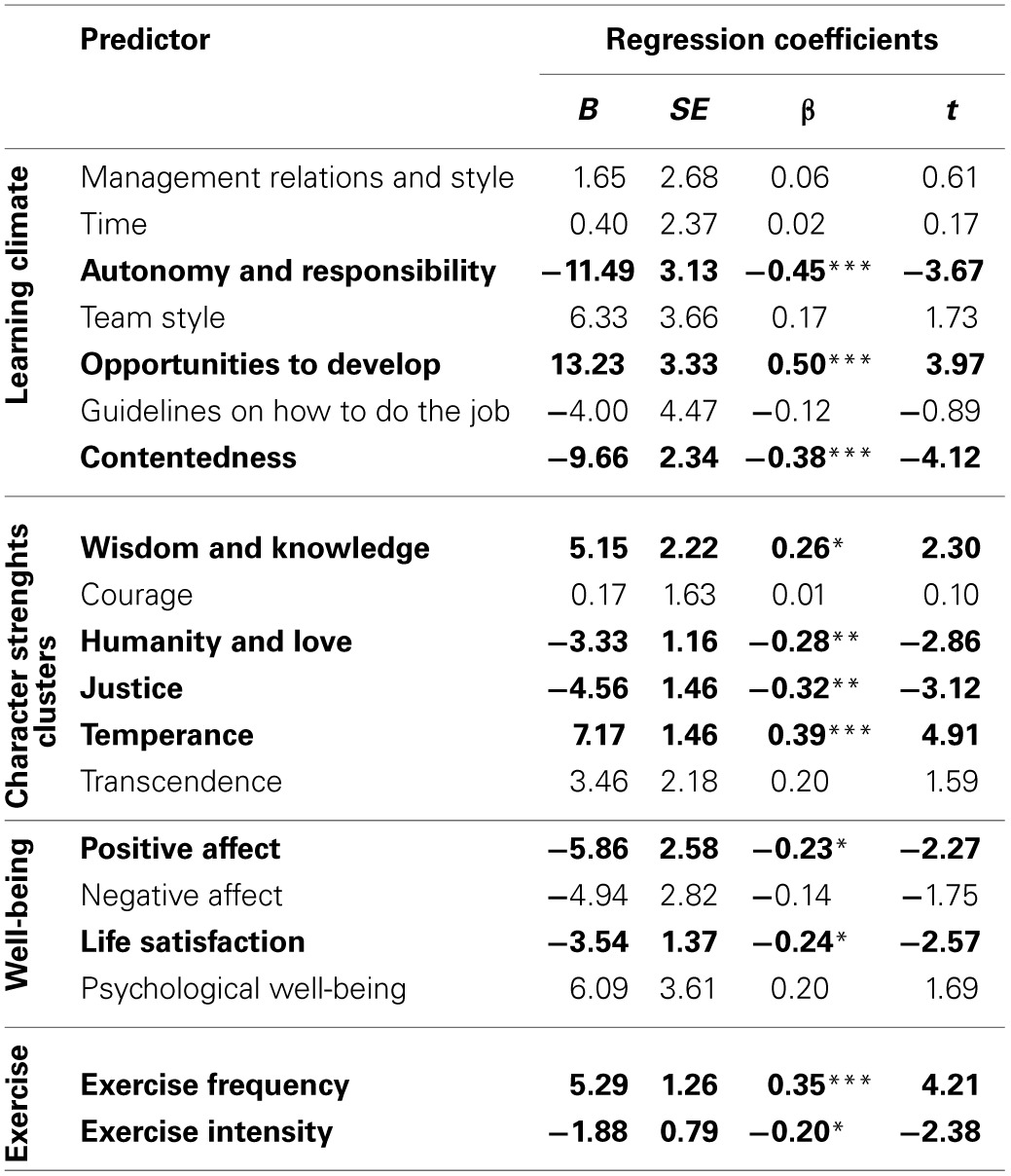
**Summary of the multiple regression analysis for learning climate variables, well-being and physical activity on the performance at work over the 6-month period**.

The model explained 55.4% of the variance in performance at work [F_*(19, 109)*_ = 5.89, p < 0.001].

Adj R^2^ = 0.46, F_(19, 90)_ = 5.89; p < 0.001; ^*^p < 0.05; ^**^p < 0.01; ^***^p < 0.001. Significant relationships highlighted in bold type.

## Discussion

The present study aimed to investigate if learning climate, character strengths clusters, well-being, and exercise habits predicted work performance over a 6 month period among call center agents. High performance was predicted by one learning climate dimension: agents' sense of having opportunities to develop at the work place (e.g., perceiving that there are different ways to learn new tasks and having opportunities to develop one's own strengths); two character strength clusters: Wisdom and Knowledge (e.g., being curious an thirsty for learning, open-minded, ingenious, social intelligent, and able to see things/problems from different angles) and Temperance (e.g., having self-control, being prudent, humble, and modest), and also by how frequently they exercised. Among the variables significantly influencing performance in a negative direction the analysis put forward contradictory results to those found in earlier research at other types of work places: autonomy and responsibility (e.g., the sense of feeling freedom to organize one's work, feeling encouraged to take responsibility and risk for one's own performance) and contentedness with the work place predicted low levels of performance over the 6-month time frame; strengths of character associated to helping others (i.e., Humanity and Love) and affiliation, fairness, and leadership (i.e., Justice) predicted low performance; the experience of positive emotions and satisfaction with life also predicted low levels of performance as well as level of exercise intensity.

The results with regard to learning climate postulate that agents' perception of the opportunities to learn new jobs and do different types of work makes them achieve more “time on the phone,” or simply spent more active time at work. Call center agents' specific job characteristics such as call-handling, repetition, monotony and noise-levels, and duration of the shifts might explain why agents strive for and embrace opportunities to learn alternative tasks or simply respond to different customer inquiries instead of repeating the same task all the time. Moreover, the hope for getting promoted or developing their skills and abilities might motivate agents to work harder, thus, being more productive and innovative (Sugrue, 2004). Moreover, the concept of Autonomy and Responsibility as part of the learning climate is quite in contrast with agents' job design of strict performance monitoring and lack of control over most aspects of their jobs. Perhaps explaining why agents' participation in decision-making and initiating actions only resulted in poor performance. Furthermore, “autocratic” decision-making processes have been supported for better productivity in more administrative tasks (Wood et al., 2013).

Oddly as it may seem, agents who were more contented about their workplace climate spend “less time on the phone.” In other words, low levels of contentment were related to high levels of performance. Considering the call center work design, sometimes labeled “the dark satanic mills of the twenty-first century” (Holman, 2003a, p. 123), this might as well be an unsatisfied worker's response in order to “Libera Te Ex Inferis” or “Free Yourself From Hell” by trying harder to reach better opportunities; this in turn, goes hand-in-hand with our rationale with regard to the positive relationship between opportunities to develop and performance. The association between satisfaction with the workplace and work performance has been fueling an unsolved hot debate sometimes called the job satisfaction-performance controversy—doubting the existence, the directness, and the direction of causality in this relationship (Greene, 1972; Wood et al., 2013, p. 63, for a comprehensive discussion). For instance, in a recent study call center agents who first reported their performance over a 6-month period and then their emotions at work for the last weeks reported experiencing more positive emotions at work that those who reported their emotions first and their performance afterwards. Suggesting that thinking about their own performance had primed them to remember having experiencing more positive emotions at work (Garcia and Archer, Under evaluation).

In regard to character strengths clusters, Wisdom and Knowledge and Temperance are the only clusters that were positively related to agents' performance. Wisdom and Knowledge comprises strengths of character such as creativity, perspective, open-mindedness and love of learning; these strengths might help agents to handle each customer; after all the call center environment does not allow teamwork, or receiving/giving support from peers. Temperance is all about protecting oneself against excesses by exerting self-control and regulating feelings and actions, and also showing prudence, humility, and modesty. As described in the introduction call center agents are monitored for each minute of their time on the phone (e.g., Garcia and Archer, 2012) and need to manage their emotional expressions toward customers (Hochschild, 1983; Holman et al., 2002). For instance, the way agents display emotions and the effort involved in managing one's emotions in exchange for remuneration has been labeled “emotional labor” (Hochschild, 1983, p. 7). Hence, in order to be productive in such an “electronic sweatshop” (Holman, 2003a) the level of self-control seems to be important—possessing and exerting Temperance at a call center might help individuals to keep away from distractions and follow their schedule, to regulate their emotions and being humble when talking to angry customers. Moreover, although the time customers spend waiting in line is important for customer satisfaction (i.e., the more time in line the less customer satisfaction), there are indications showing that the information received and the way the customer has been treated is more important than time in line, especially for customers waiting great amounts of time (Garcia et al., 2012b). If so, besides a humble and self-controlling agent, customers might appreciate and receive more time from agents who exert the characters strengths included in the Wisdom and Knowledge cluster. Thus, perhaps explaining why this character strength cluster predicted high performance—agents high in these character strengths might expend more time explaining and/or searching for information while having the customer at the other end of the phone, which at the end means more “time on the phone” for the agent.

Also in this vein, character strengths clusters like Humanity and Love and Justice that are communal values, such as kindness and generosity, equity, and teamwork, are understandably related to low levels of performance. Celebrating, possessing and willing to exercise these virtues in a workplace that does not give opportunities for teamwork, organizing group activities and socializing with others may only add to thwarting the agents' feelings and consequently deteriorate their performance level. Also, spending time exerting communal values might lead to more time expending helping colleagues or trying to help customers beyond what is possible, thus, reducing the time agents spend on the phone.

The negative associations between performance and life satisfaction and positive affect simply suggest that agents reporting higher subjective well-being at the beginning of the study has resulted in lower performance during the 6-month period. This adverse influence of well-being on performance may be explained by the definition of subjective well-being, call centers' specific work-design, and expected performance criteria in a call center. Philosophy of hedonism considers pleasure as the only good thing for us (Forgeard et al., 2011) and suggests “the pleasant life” (pursuing pleasant emotions life) as the pathway toward happiness (Kristjánsson, 2010). Individuals experiencing higher levels of subjective well-being may find acting as a call center agent to be an unpleasant activity that leaves no chance to express, practice or experience pleasure at work. Hereafter it seems more understandable for them to put less effort into an unpleasant activity, thus, leading to lower work performance. Nevertheless, all positive measures of well-being (i.e., positive affect, life satisfaction, and psychological well-being) were related to the majority of the learning climate variables and character strengths clusters (see also Archer and Garcia, 2014; Archer and Garcia, who showed that subjective well-being is positively related to academic performance). In other words, well-being's relationship to performance might be a function of different learning climate and character strengths.

Finally, frequent physical activity predicted performance, while level of intensity of physical activity was negatively related to it. Beneficial effects of frequent physical activities on different aspects of physical and mental health and well-being are well-understood (Fuchs, 2001; Schlicht, 2001; Teychenne et al., 2008; Garcia et al., 2012a). Regular exercising has been shown to have positive influence on several workplace outcomes such as performance, absenteeism and work productivity (Frigeri, 2010; Barr-Anderson et al., 2011; Arvidson et al., 2013), perhaps because frequent exercise reduces stress symptoms and improves mental states, and in the long term, enable arousal levels to be more appropriate adjusted for cognitive work and by increased stress resistance (Garcia et al., 2012b; Archer and Garcia, 2014). Frequent physical activity, for instance, was associated to the character strengths cluster of Wisdom and Knowledge; which comprises strengths of character needed in cognitive work and that were related to high performance in the present study. Conversely, high intensity of physical activity might give call center agents more strain than alleviation to their already strained working conditions. Indeed, previous studies show inconsistent results regarding the exercise intensity and its positive consequences (Salmon et al., 2003; Teychenne et al., 2008; Asztalos et al., 2010; Frigeri, 2010; Kirk and Rhodes, 2011)—academic performance, for instance, is related to intensity not frequency of physical activity (Archer and Garcia, 2014).

### Limitations and suggestions for future research

The first limitation of this study may be related to the sample size that was relatively small. The performance measure used here, and in most call centers for that matter, accounts for agents' “time on phone,” which is seen as the most important factor in determining customers' “queue time”; a factor that in turn is directly linked to customer's satisfaction level (Davis and Volmann, 1990; Durrande-Moreau, 1999). Indeed, some call center managers even define a “magic actual time” that when transgressed, leads to customer dissatisfaction (Garcia et al., 2012b). Yet, satisfaction with the information received and the way the customer has been treated by an agent has been shown to be among the most important factors for customer satisfaction (Garcia et al., 2012a,b). In their study including 5851 call center customers, Garcia et al. (2012a,b) concluded that, in fact, the information received and the way agents treated them are the dominant factors in determining customers' satisfaction level. Providing satisfactory information and behaving openly with customers as other performance indices need then to be addressed. Doing so might lead to different results that those found here with regard to learning climate, character strengths, and well-being.

In addition to the explanations given above with regard to well-being, it should not be overlooked that positivity (i.e., emphasizing the importance of positive notions such as life satisfaction, positive affect, flow, hope, optimism, virtues, and so on), as the heart of positive psychology movement (Seligman and Csikszentmihalyi, 2000) and other pertinent research themes such as positive organizational scholarship (Cameron et al., 2003) and positive organizational behavior (Luthans, 2002), has received criticism because of their “… implicit acceptance of fundamental flaws in how work and organizations are designed” (Hackman, 2009, p. 309). Hence, findings in this research vein may not be taken for granted without considering specific work settings (see for example Garcia et al., 2014b, who tested the validity of a personality instrument designed for work force recruitment). Moreover, employees' perception of performance monitoring may have a moderation effect on the association between their well-being and their actual performance. Further investigations are definitely needed to disentangle the confusion raised from our findings.

### Practical implications

These findings denoted several practical opportunities for call center managers to enhance their agents' performance. First, improvement of work climate in call centers may need to include “opportunities to develop” as a decisive factor in keeping agents effortful to reach higher or more specialty-based positions in the organization. Second, human resources authorities in a call center should pay more attention to individual differences in the recruitment and selection procedures of call center agents to prevent future shortcomings or worker dissatisfaction. Nevertheless, the performance appraisal in call centers may need to go through a reconsideration process to include other aspects of successful performance that might be more attuned with different character strengths and individuals' happiness (i.e., life satisfaction and positive emotions). After all, allowing individuals to put their strengths at work should empower the individual and at the end the organization itself. Finally, physical activity enhancement programs, whether designed as a work routine activity of an agent or to be performed and rewarded in her/his leisure time, might offer considerable positive work outcomes.

“There is no greater sorrow

     than to recall happiness in

     times of misery”

     Dante Alighieri

### Conflict of interest statement

The authors declare that the research was conducted in the absence of any commercial or financial relationships that could be construed as a potential conflict of interest.
